# Exploration of an *Actin* Promoter-Based Transient Expression Vector to Trace the Cellular Localization of Nucleorhabdovirus Proteins in Leafhopper Cultured Cells

**DOI:** 10.3389/fmicb.2018.03034

**Published:** 2018-12-19

**Authors:** Xiao-Feng Zhang, Yunjie Xie, Haitao Wang, Juan Wang, Hongyan Chen, Tianbao Zeng, Yibing Zhao, Taiyun Wei

**Affiliations:** State Key Laboratory of Ecological Pest Control for Fujian and Taiwan Crops, Institute of Plant Virology, Fujian Agriculture and Forestry University, Fuzhou, China

**Keywords:** *actin* promoter, transient expression vector, insect cultured cell, RYSV P6, viroplasm

## Abstract

Continuously cultured cell lines derived from planthopper and leafhopper have greatly facilitated the investigation of rice viruses transmitted by these insects. However, the lack of a suitable transient expression vector has limited their utility. Here, by cloning and analyzing the promoter sequence of the gene encoding cytoplasmic actin from the leafhopper *Nephotettix cincticeps*, we successfully developed the first efficient transient expression vector for cultured leafhopper cells, which can also be used to express exogenous proteins in other insect culture cell lines, including those derived from *Recilia dorsalis* leafhopper and *Spodoptera frugiperda* (Sf9). Furthermore, insertion of the *Hr5* viral enhancer element and knockdown of the endogenous *Dicer2* gene notably improved the vector’s expression efficiency in leafhopper cells. Using the optimized vector, we have for the first time traced the cellular localization of the proteins encoded by rice yellow stunt virus (RYSV) in cells of its insect vector and demonstrated that P6 protein is a component of the viroplasm.

## Introduction

Planthoppers (Hemiptera: Fulgoromorpha) and leafhoppers (Hemiptera: Cicadellidae) are the most destructive insect pests of rice in the temperate and tropical regions of East and Southeast Asia. They not only cause direct detrimental effects on plant growth, such as plant wilting and leaf chlorosis but also transmit several rice pathogens, including phytoplasma and plant viruses, that cause enormous economic loss every year (Oya, 1980; Sōgawa, 1982). Rice-infecting rhabdoviruses are transmitted in a persistent manner by leafhoppers (Jackson et al., 2005; Wei and Li, 2016; Yang et al., 2017; Wang et al., 2018b). These viruses possess complex genomes comprising a long negative-strand single-stranded RNA (-ssRNA) and have complicated infection strategies (Dietzgen et al., 2017; Lefkowitz et al., 2018). These features increase the difficulty of studying these viruses in both their host plants and insect vectors. Moreover, the limited number of technical innovations in insect systems thus far has seriously hampered research progress.

The first continuously cultured leafhopper cell line was established in 1964 (Hirumi and Maramorosch, 1964), and major improvements over the original cultured cells in the succeeding decades have made these cells an important tool for studying the behavior of rice viruses in their vector cells, including during viral entry, replication, and spread (Wei et al., 2006a,b; Ma et al., 2013; Chen et al., 2015; Zheng et al., 2015; Jia et al., 2017). To date, over 100 publications have been related to applications of leafhopper cultured cells. Despite this progress, no expression systems have been developed for cultured leafhopper cell lines, which have greatly limited the investigation of rice viruses.

Several systems are available for exogenous protein expression in other types of insect cells. These systems are divided into two major categories: 1) virus-based expression systems of which the baculovirus expression vector (BEV) system is the most successful, with thousands of recombinant proteins produced so far (van Oers et al., 2015) and 2) viral gene promoter- or endogenous gene promoter-based expression vectors among which the lepidopteran nucleopolyhedrovirus *immediately early 1* (*IE1*) gene promoter fused with the *homologous region 5* (*Hr5*) enhancer (Hr5/IE1) is a successful example (Viswanathan et al., 2003; Lin et al., 2010). Unfortunately, none of these existing expression systems works in cultured leafhopper cells. The development of a leafhopper transient expression vector is a pressing need for the investigation of rice viruses and will considerably expand the research applications of cultured leafhopper cell lines.

Rice yellow stunt virus (RYSV), first reported in 1965 in Taiwan and southern China (Chiu et al., 1965), is the only rice-infecting nucleorhabdovirus in the family *Rhabdoviridae*; it is persistently transmitted by the leafhopper *Nephotettix cincticeps* (Hemiptera: Delphacidae) and caused major reductions in rice yields in southern China from the 1970s to the 1980s (Faan and Pui, 1980; Wang et al., 2018a,b). The genome of RYSV is a single negative-strand RNA of 14,030 nucleotides that harbor seven open reading frames encoding seven proteins: nucleoprotein (N), phosphoprotein (P), movement protein (P3), matrix protein (M), glycoprotein (G), RNA silencing suppressor P6, and large RNA polymerase (L), in the order 3′-N-P-3-M-G-6-L-5′. The functions and cellular localizations of the plant rhabdovirus-encoded proteins in plant hosts have been extensively investigated for Sonchus yellow net virus (SYNV) (Goodin et al., 2007), Potato yellow dwarf virus (PYDV) (Bandyopadhyay et al., 2010), and RYSV (Fang et al., 1994; Zhu, 1997; Luo et al., 1998; Luo and Fang, 1998; Huang et al., 2003, 2005). However, less work has been done on the behavior of these viruses in their insect vectors. In particular, the lacks of suitable insect cell cultures and transient expression vectors have made it difficult to trace the infection of plant rhabdoviruses in cultured insect cells.

In this study, we analyzed a leafhopper *cytoplasmic actin* promoter and successfully used it to develop a transient expression vector, which was able to drive expression of green fluorescent protein (GFP) in several insect cell culture lines, including different leafhopper cell lines and Sf9 cells. We then optimized this vector by inserting the *Hr5* enhancer and found that knocking down *Dicer2* to inhibit RNA interference (RNAi) also improved the expression efficiency of the construct. We used our new expression system to detect the cellular localizations of the RYSV proteins in leafhopper cells and demonstrated that the P6 protein of RYSV is a component of the RYSV viroplasm.

## Materials and Methods

### Cell Culture and Reagents

Continuous monolayer cultures of vector cells, including *N. cincticeps*, *Nephotettix apicalis*, *Recilia dorsalis*, and Sf9, were prepared as previously described (Ma et al., 2013; Chen et al., 2014). Leafhopper cells were grown on coverslips (diameter 15 mm) to 80% confluence and then were inoculated with 50 μl of viral inoculum prepared from RYSV-infected rice leaves as previously described (Wei et al., 2006b). The infected cells on coverslips were then subjected to an immunofluorescence assay with appropriate antibodies. Antibodies against the RYSV proteins were prepared as previously described (Wei et al., 2007; Jia et al., 2012; Wang et al., 2018b). IgGs were purified from the respective protein-specific polyclonal antibodies and conjugated directly to fluorescein isothiocyanate (FITC) or rhodamine according to the manufacturer’s instructions.

### Construction of Expression Vectors

All vectors used in this study were derived from the vector pEGFP-N3 (GenBank: U57609.1). The sequences of putative promoters, donated as a gift from Dr. Yi Li of Peking University, were amplified from genomic DNA by PCR with the specific primers listed in Table S3, which introduced *Eco*RI and *Bam*HI restriction enzyme sites at the 5′ and 3′ ends of the PCR fragments. The purified PCR products were then cloned into the multiple cloning site (MCS) of pEGFP-N3 with *Eco*RI and *Bam*HI. The original cytomegalovirus (CMV) promoter was removed by digestion with *Vsp*I and *Eco*RI. The resulting plasmid was named NC-Cyto-GFP. To construct the plasmid NC-Hr5Cyto-GFP, the *Hr5* enhancer element, amplified from pHr5/IE1-EGFP (GenBank: AF402295.1) by PCR with specific primers (F-Hr5/R-Hr5 in Table S1), was ligated into *Eco*RI-digested NC-Cyto-GFP using the Gibson assembly cloning kit (catalog # E5510s, NEB).

The gene fragments encoding RYSV proteins (*N*, *P*, *P3*, *M*, *G*, and *P6*) without *L* due to its long length (over 6000 bp) were amplified with specific primers (listed in Table S1) and integrated into *Bam*HI-digested NC-Hr5Cyto-GFP using the Gibson assembly cloning kit to obtain vectors expressing the fusion proteins N-GFP, P-GFP, P3-GFP, M-GFP, G-GFP, and P6-GFP, respectively, where the C-terminus of the viral protein is fused to the N-terminus of GFP.

To construct the plasmids expressing P6-His, N-Strep, and P-Strep in *N. cincticeps* cells, sequences encoding a 6× His tag or a Strep tag were introduced just before the original stop codons of the *P6* gene and the *N* and *P* genes, respectively. The resulting fragments were then cloned into NC-Hr5Cyto-GFP via *Bam*HI and *Sma*I sites to take the place of the GFP fragments. The resulting vectors were named NC-Hr5Cyto-P6-His, NC-Hr5Cyto-N-Strep, and NC-Hr5Cyto-P-Strep.

### Plasmid DNA Transfection Assay

Four cultured insect cell lines including *N. cincticeps, N. apicalis, R. dorsalis*, and Sf9 cells were transfected with the Cellfectin® II Reagent (catalog # 10362100, Thermo Fisher Scientific), Lip2000 (catalog # 11668027, Invitrogen), and Lip3000 (catalog # L3000001, Invitrogen) following the manufacturer's instructions. Different ratios of plasmid to Cellfectin® II were transfected to obtain the best expression efficiency of the candidate gene. About 2 μg plasmid mixed with 4 μl Cellfectin® II provided the best efficiency of protein expression in *N. cincticeps* cultured cells. All experiments were conducted in biological repeats.

### Immunofluorescence Microscopy

RYSV-infected vector cell monolayers (VCMs) or plasmid-transfected cells on glass coverslips were fixed at 6 days post inoculation (dpi) in 4% v/v paraformaldehyde in 0.01 M phosphate-buffered saline (PBS) buffer at room temperature for 2 h and then permeabilized in 0.2% Triton X-100 at room temperature for 30 min. Cells were subjected to incubation with a 100-fold-diluted solution of directly conjugated IgG. Samples were observed with a Leica TCS SP5 inverted confocal microscope, as described previously (Zheng et al., 2015).

### Transmission Electron Microscopy

The RYSV-infected cultured leafhopper cells (46 sections) were embedded in Spurr resin medium and used to prepare ultrathin sections, as described previously (Wang et al., 2018b). The block of VCM samples was cut into slices with an ultramicrotome (Leica UC7), incubated with N-specific or P6-specific IgGs, and then labeled with goat antibodies to rabbit IgG conjugated with 15-nm gold particles or goat antibodies to mouse IgG conjugated with 10-nm gold particles (Sigma), as previously described (Wang et al., 2018b). The labeled ultrathin sections were observed by electron microscopy.

### Yeast Two-Hybrid Assay

The yeast two-hybrid assay and the Matchmaker Gal4 Two-Hybrid system 3 (Clontech) were used to detect the interaction between the RYSV P6 protein and the N and P proteins. The full-length *N*, *P*, and *P6* gene fragments were amplified and cloned into the bait vector pGBKT7 and the prey vector pGADT7, respectively. All the target constructs were sequenced. Combinations of bait and prey plasmids (pGBKT7-P6/pGADT7-N and pGBKT7-P6/pGADT7-P) were co-transformed into the AH109 yeast strain and grown on SD/-Leu/-Trp/-His/-Ade/X-a-gal culture medium. The combination pGBKT7-53/pGADT7-T served as a positive control and the combinations pGBKT7-Lam/pGADT7-T, pGBKT7-N/pGADT7, and pGBKT7/pGADT7-P as negative controls.

### Knockdown of 3′Utr and P6 Gene During Rysv Infection By Transfection of the *in vitro*-Synthesized dsRNAs Into Vcms

Double-stranded RNAs of the 3′ untranslated region (ds3′UTR) and the *P6* gene (dsP6) for RNAi were prepared according to the manufacturer’s instructions as previously described (Lan et al., 2016; Mao et al., 2017). The double-stranded RNAs were transfected into VCMs with Cellfectin II (Thermo Fisher Scientific, Catalog # 10362100) following the manufacturer’s instructions. At 8 h after the dsRNA treatments, the VCMs were incubated with RYSV inoculum for 2 h and cultured for 72 h post inoculation (hpi), after which they were subjected to an immunofluorescence assay with an antibody specific to N or P (Wang et al., 2018b). RT-qPCR was also used to detect the relative transcript levels of the *N*, *M*, and *P6* genes (primers are described in additional file 1) against the transcript level of leafhopper actin gene to verify the effects of the RNAi and the RYSV infection. RT-qPCR assays were conducted in triplicate and analyzed using Student’s *t*-test.

## Results

### Construction and Evaluation of *N. cincticeps Cytoplasmi*c *Actin* Promoter-Based Vector in Four Different Insect Cultured Cell Lines

**Figure 1:**
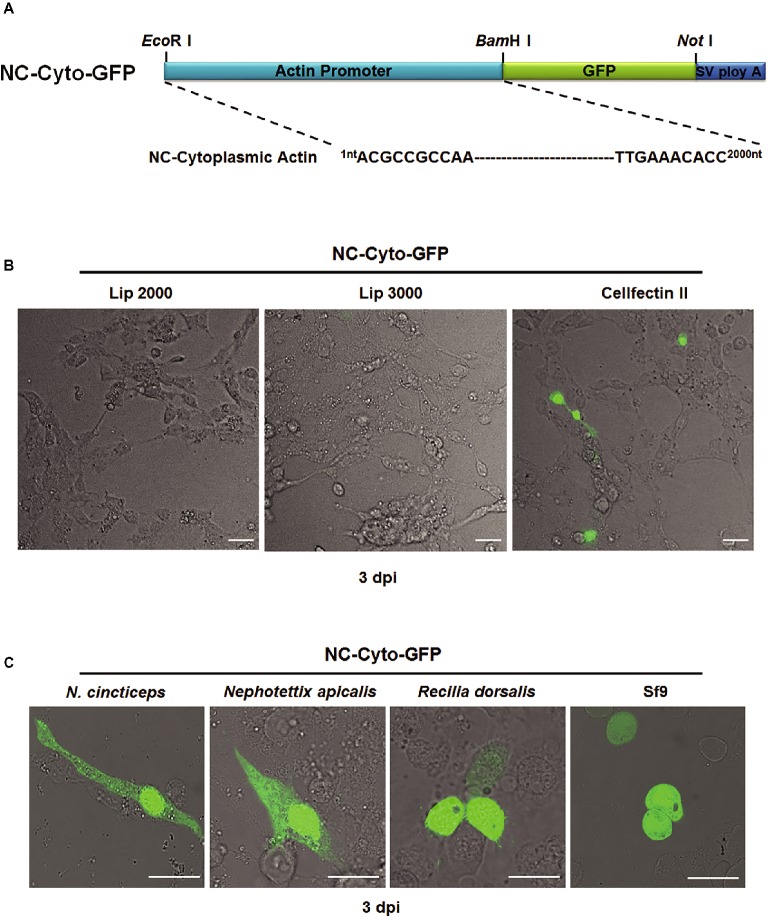
Evaluation of the efficiency of the *N. cincticeps cytoplasmic actin* promoter in *N. cincticeps*, *N. apicalis*, *R. dorsalis*, and Sf9 cells. **(A)** Schematic representation of the NC-Cyto-GFP construct. **(B)** Cultured *N. cincticeps* cells transfected with NC-Cyto-GFP via different liposome reagents observed 3 days post transfection. **(C)** Various insect cell culture lines, including *N. cincticeps*, *N. apicalis*, *R. dorsalis*, and Sf9 cells, transfected with NC-Cyto-GFP and observed 3 days post transfection. Scale bar: 20 μm. SV poly A indicates Simian vacuolating virus 40 poly A sequence.

To construct the leafhopper *cytoplasmic actin* gene promoter-driven expression vector, we amplified 2000 base pairs of nucleotide sequence just upstream from the start codon of the gene encoding cytoplasmic actin from the *N. cincticeps* leafhopper genome using specific primers (Table S3). The resulting fragment (considered to be the candidate promoter) was integrated into the plasmid pEGFP-N3, replacing the CMV promoter, by *Eco*RI and *Bam*HI digestion. We then mixed the recombinant plasmid, which we named NC-Cyto-GFP (Figure 1A), with one of three different commercial liposome preparations, Cellfectin II, Lip2000, and Lip3000, and transfected it into cultured *N. cincticeps* cells. Three days after transfection, only the cultured cells transfected with the mixture of NC-Cyto-GFP and Cellfectin II showed a positive GFP signal (Figure 1B), indicating that the promoter of the *cytoplasmic actin* gene functioned to direct the expression of the exogenous *GFP* gene in the cultured cells. We therefore used Cellfectin II in later experiments.

Next, to obtain a higher transfection efficiency, we optimized the ratio of plasmid (μg) to Cellfectin II (μl) in the transfection mixture by adding different amounts of plasmid DNA, ranging from 1 to 6 μg, to 4 μl of Cellfectin II diluted according to the manufacturer's instructions. Mixing 4 μl Cellfectin II with 2 μg plasmid provided the best transfection efficiency (about 5%) in leafhopper cells (Table S1). We also assessed the inoculation time and determined that the proportion of leafhopper cells containing GFP reached its maximum at 48 h post transfection and did not decrease even at 96 h post transfection (Table S2).

We then evaluated our new expression cassette in cultured cell lines from several different insects, including *N. cincticeps, N. apicalis, R. dorsalis*, and Sf9 cells. At 48 h post transfection under optimal conditions, we observed GFP signals in all tested cell lines, but the transfection efficiency remained low, with the maximum being around 10% in Sf9 cells (Figure 1C).

### Optimization of Transient Expression Vector in Cultured *N. cincticeps* Cells

**Figure 2:**
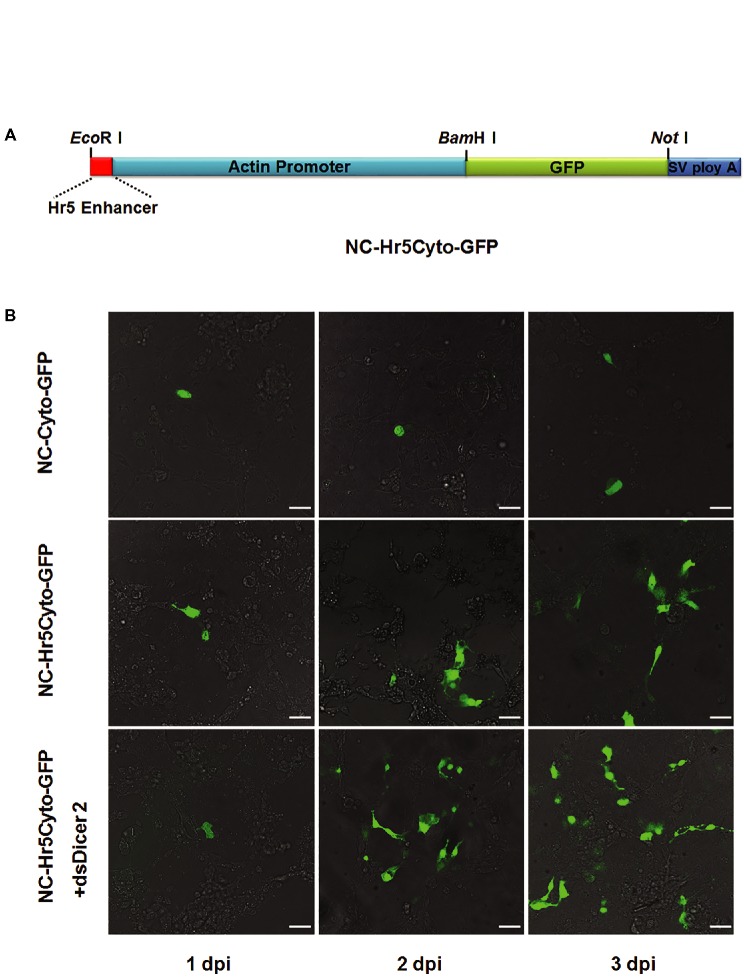
Optimization of *N. cincticeps cytoplasmic actin* promoter efficiency in *N. cincticeps* cells. **(A)** Schematic representations of the NC-Hr5Cyto-GFP construct. **(B)** Examination of the optimized vector NC-Hr5Cyto-GFP in cultured *N. cincticeps* cells. Cells transfected with the NC-Cyto-GFP vector were used as a control. dsDicer2 RNA was co-transfected with NC-Hr5Cyto-GFP. Scale bar: 20 μm.

The low expression efficiency (5%) that we obtained in *N. cincticeps* cells was insufficient for our needs in further experiments. To optimize the transient expression vector, we incorporated the *Hr5* enhancer sequence just upstream of the promoter sequence of the *cytoplasmic actin* gene to obtain a new expression vector, NC-Hr5Cyto-GFP (Figure 2A). We then transfected NC-Hr5Cyto-GFP mixed with Cellfectin II into *N. cincticeps* cells. At 48 hpi, as compared with NC-Cyto-GFP-transfected control cells, the NC-Hr5Cyto-GFP-transfected leafhopper cells showed more GFP fluorescence signals (around 10% of cells fluoresced) (Figure 2B).

To further improve the expression efficiency of the target protein in *N. cincticeps* cells, we tried knocking down the endogenous *Dicer2* gene during the transient transfection. We co-transfected 1 μg *dsDicer2* RNA into *N. cincticeps* cells along with the 2 μg NC-Hr5Cyto-GFP mixed with Cellfectin II (4 μl). At 3 dpi, around 30% of transfected cells showed green fluorescence (Figure 2B). This result suggested that RNA silencing in *N. cincticeps* cells can affect the expression of exogenous genes and that knockdown of *Dicer2* improves the transfection efficiency of such genes.

### Cellular Localization of Transiently Expressed Rysv-Encoded Proteins in *N. cincticeps* Cells

**Figure 3:**
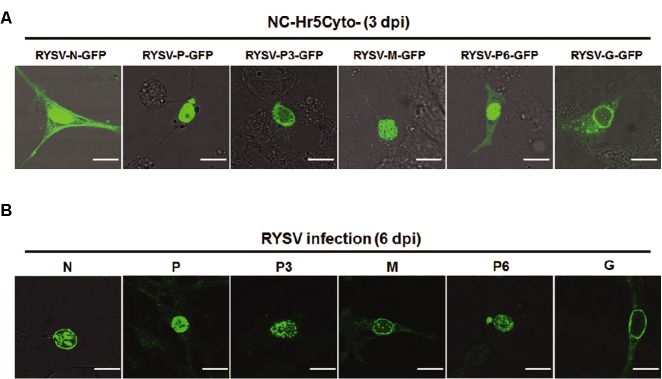
Laser-scanning confocal micrographs showing the subcellular localization of RSYV-encoded proteins in *N. cincticeps* cells. **(A)** Fluorescence of *N. cincticeps* cells expressing GFP fusions of different RYSV-encoded proteins. Scale bar: 20 μm. **(B)** Confocal immunofluorescence micrographs showed the cellular localization of RYSV proteins in RYSV-infected *N. cincticeps* cells. Infected VCMs at 6 dpi after RYSV inoculation, immunolabeled with viral-specific IgGs directly conjugated to FITC. Scale bar: 20 μm.

To determine the ability of the optimized vector to express exogenous protein in leafhopper cells, we inserted six RYSV genes, *N*, *P*, *P3*, *M*, *G*, and *P6*, into the NC-Hr5Cyto-GFP plasmid directly downstream from the promoter sequence followed by the *GFP* gene fragment. We transfected the resulting recombined vectors expressing viral proteins fused with GFP into cultured *N. cincticeps* cells via the optimized protocol. At 3 dpi, we inspected slices of cultured *N. cincticeps* cells transfected with the different expression cassettes by confocal fluorescence microscopy. The microscopy observations indicated that transiently expressed most N-GFP, P-GFP, M-GFP, and P6-GFP were all localized in the nucleus, while a few N-GFP and P3-GFP accumulated in the cytoplasm. More interestingly, G-GFP, the envelope glycoprotein of the RYSV virion, was arrayed around the nuclear membrane (Figure 3).

To verify the results of the transient expression assay, we investigated the subcellular localization of the RYSV-encoded proteins during viral infection via immunofluorescence assays. At 6 dpi, we fixed RYSV-infected cells and incubated them with antibodies specific to the different RYSV-encoded proteins. The subcellular localizations of N, P, G, and P6 were consistent with what we had observed in the transient expression assay, whereas M proteins were detected around the nuclear membrane, indicating that GFP may affect the localization of the M protein in the transient expression assay. Meanwhile, the P3 protein accumulated in the nuclei of the infected cells (Figure 3B). As a member of the genus *Nucleorhabdovirus*, RYSV is believed to assemble its viroplasm in the nuclei of the host cell. Our transient expression results thus showed that P6, N, and P, which have been demonstrated to be components of the RYSV viroplasm, were all localized in the nucleus. This result inspired us to study whether RYSV P6 contributes to the assembly of viroplasm.

### P6 Protein Forms a Viroplasm-like Structure in the Nuclei of RYSV-infected Leafhopper Cells

**Figure 4:**
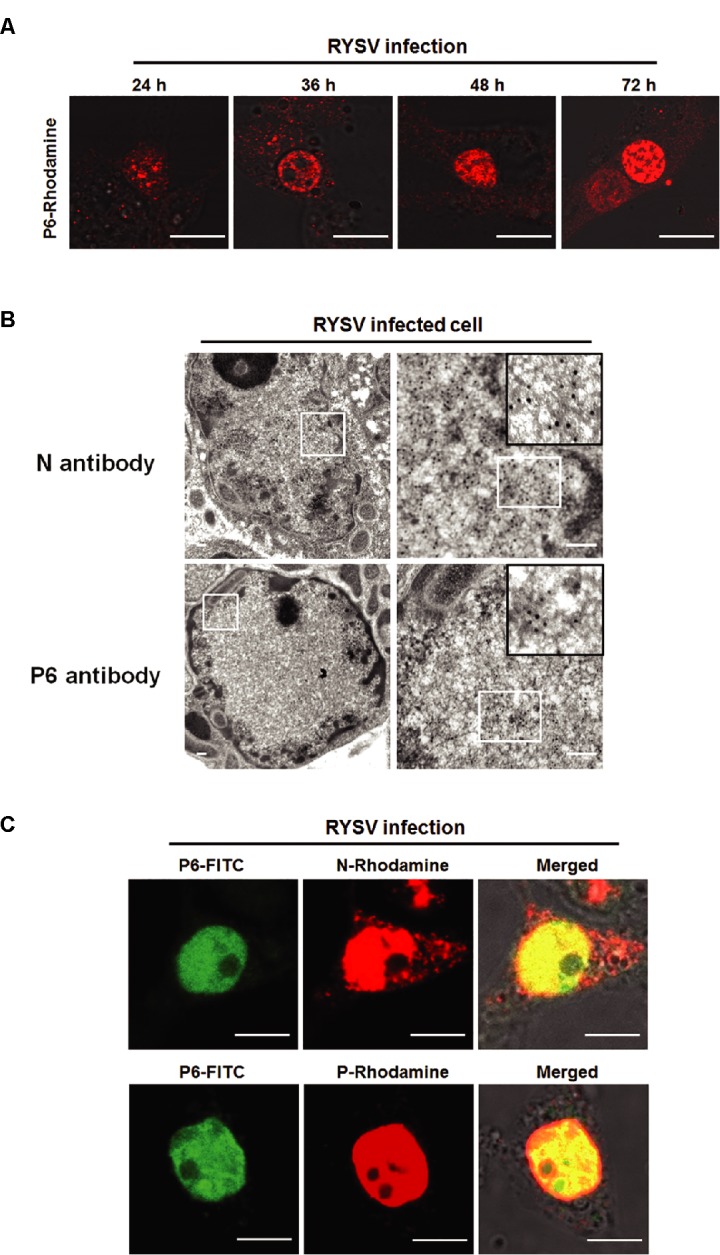
Subcellular localization of P6 proteins of RYSV in viral-infected VCMs. **(A)** Morphogenesis of RYSV P6-formed structure in the nucleus of the RYSV-infected *N. cincticeps* cell at different time points of RYSV infection. VCMs infected by RYSV were labeled with antibody specific to RYSV P6. Scale bar: 20 μm. **(B)** Electron micrographs showing subcellular localization of RYSV P6 in RYSV-infected VCMs. Samples were immunolabeled with P6-specific polyclonal antibodies as primary antibodies and then treated with goat-anti-rabbit IgG conjugated to 15-nm-diameter gold particles as secondary antibodies. RYSV-infected VCMs were labeled with N-specific antibody separately, as a control. Scale bar: 100 nm. **(C)** Confocal immunofluorescence micrographs showing the colocalization of the P6 protein and the N and P proteins in the RYSV-infected *N. cincticeps* cell. Scale bar: 20 μm.

To investigate the potential function of P6 in the nucleus, we detected the subcellular distribution of P6 during RYSV infection via an immunofluorescence assay. We first labeled the RYSV-infected cultured *N. cincticeps* cell with antibodies specific to P6 or P6-rhodamine (P6-R) and then subjected them to confocal microscope observation at different time points after viral infection. At 24 hpi, a small amount of P6 protein coalesced into tiny, intensely fluorescent foci scattered inside the nucleus. At 36 hpi, the foci underwent dynamic changes in shape, size, and quantity. During the late stage of RYSV infection, at 72 hpi, the large aggregates formed by P6 almost filled the entire nuclei of the cells (Figure 4A). The morphometric changes of P6-formed structures during RYSV infection were identical to those of VCMs formed by N proteins and P proteins, which we previously described (Wang et al., 2018a,b). Our transient expression results thus showed that N and P proteins, which have been demonstrated to be components of the RYSV viroplasm (Wang et al., 2018a,b) as well as P6 protein, were all localized in the nucleus.

To further clarify the role of P6 in the assembly of RYSV viroplasm, we observed the aggregates formed by P6 in the nucleus via an immunogold labeling assay. We treated the cells with rabbit antibodies specific to P6, as primary antibodies, followed by goat-anti-rabbit IgG that had been conjugated with 15-nm-diameter gold particles, as secondary antibodies. Ultrathin sections of RYSV-infected VCMs were also labeled with N-specific antibody and 15-nm-diameter gold particles as a control. Electron microscopy observations showed that the gold particles representing P6 proteins were specifically localized into the viroplasm-like complex, which further indicated that P6 protein is associated with RYSV viroplasm (Figure 4B).

Since we had previously demonstrated that N and P are components of the RYSV viroplasm, we next assessed the colocalization of the P6 protein with the N and/or P protein during RYSV infection. At 48 hpi, we fixed the RYSV-infected VCMs and labeled separate samples with P6 (P6-FITC)/N (N-rhodamine) antibodies and P6 (P6-FITC)/P (P-rhodamine) antibodies. Immunofluorescence microscopy results showed that the P6 protein was colocalized perfectly with the N and P proteins, filling almost the entire nucleus of RYSV-infected *N. cincticeps* cells (Figure 4C).

In summary, the above results indicated that P6 is involved in the formation of viroplasm structures in the nuclei of virus-infected insect cells.

### *In vitro* Interaction of P6, N, and P

**Figure 5:**
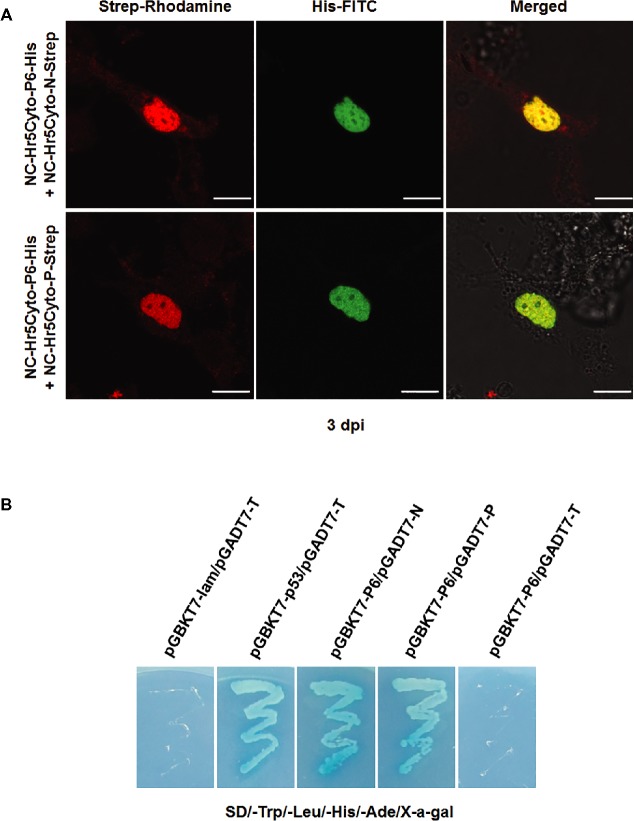
Colocalization and interactions of RYSV P6 with N and P *in vivo* and *in vitro*. **(A)** P6-His/N-Strep and P6-His/P-Strep were coexpressed in the *N. cincticeps* cell using the corresponding plasmids, while the *Dicer2* gene was knocked down by dsRNA treatment. The VCM samples were fixed and inoculated with antibodies to the His and Strep tags. Scale bar: 20 μm. **(B)** Yeast two-hybrid assay for the interactions of P6 with N and P6 with P. The transformed cells were grown on SD/-Trp/-Leu/-His/-Ade solid medium supplemented with 40 μg/ml X-α-Gal. pGBKT7-53 and pGADT7-T were used as the positive control. Yeast cells co-transformed with pGBKT7-Lam/pGADT7-T, pGBKT7-N/pGADT7, and pGBKT7/pGADT7-P were used as negative controls.

The colocalization of P6 with N and P proteins pointed to possible interactions of P6 with N and/or P. To confirm these potential interactions, we assembled the construct Nc-Hr5Cyto-P6-His, which was expected to express the recombined protein P6-His, by amplifying *P6* gene fragments fused with a 6× His tag sequence at the 3′ end and inserting it into the NC-Hr5Cyto-GFP vector through the *Bam*HI/*Sma*I enzyme sites to take the place of the GFP fragment. The plasmids NC-Hr5Cyto-N-Strep and NC-Hr5Cyto-P-Strep, expressing N-Strep and P-Strep fusion proteins, respectively, were cloned by the same method. We then mixed NC-Hr5Cyto-P6-His/NC-Hr5Cyto-N-Strep and NC-Hr5Cyto-P6-His/NC-Hr5Cyto-P-Strep and transfected them separately into cultured *N. cincticeps* cells with Cellfectin reagent. At 3 dpi, we fixed the transfected cells, labeled them with His-specific and Strep-specific antibodies conjugated directly to FITC and rhodamine (His-FITC, N-rhodamine, and P-rhodamine), and subjected them to immunofluorescence microscopy. About 10% of the transfected cells showed both green and red fluorescence, with P6-His/N-Strep and P6-His/P-Strep coexpressed and colocalized in the nucleus (Figure 5A).

To further investigate the proposed interaction between the P6 protein and the N and P proteins, we performed a yeast two-hybrid assay. As expected, only yeast cells containing the plasmids pGBKT7-53pGADT7-T (positive control), pGBKT7-P6/pGADT7-N, or pGBKT7-P6/pGADT7-P grew on the SD/-Trp/-Leu/-His/-Ade plate, and the negative control groups (pGBKT7-Lam/pGADT7-T) did not (Figure 5B). These results demonstrated that P6 strongly interacted with the RYSV viroplasm components N and P. We also looked for interactions between P6 and other RYSV-encoded proteins, P3, M, and G, via a yeast hybridization assay but found no evidence of interactions between these proteins and P6 proteins (data not shown).

### *P6* Gene Knockdown Affects RYSV Infection in Cultured *N. cincticeps* Cells

**Figure 6:**
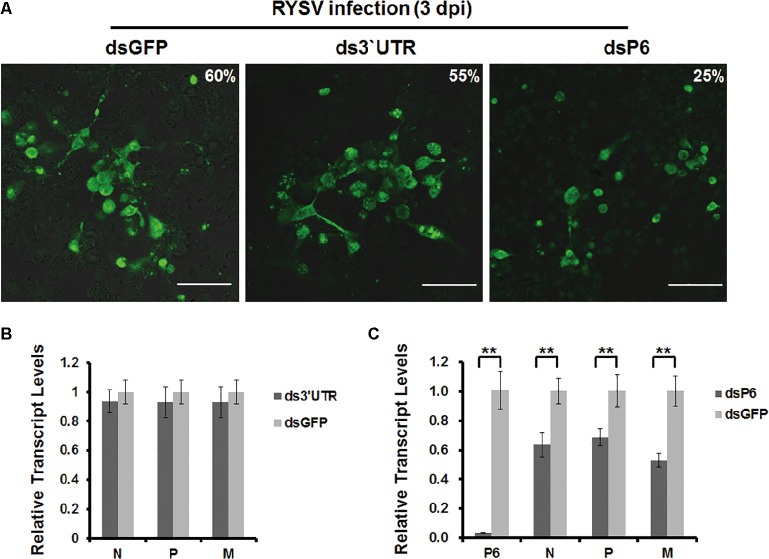
Knockdown of P6 by dsRNA treatment affects RYSV infection. **(A)** VCMs were first transfected with *dsGFP*, *ds3′UTR*, or *dsP6* and then inoculated with RYSV. At 72 h post RYSV inoculation, cells were immunolabeled with antibodies specific to N protein and subjected to confocal microscope observation. Scale bar: 25 μm. A total of 1,200 cells/condition were counted. **(B)** qRT-PCR analysis of RYSV *N*, *P*, and *M* gene transcripts after treatment with *ds3′UTR* treatment. The RYSV-infected cells were also treated with *dsGFP* as the control. **(C)** qRT-PCR analysis of RYSV *N*, *P*, and *M* gene transcripts after treatment with *dsP6* and *dsGFP*. Three biological repeats were done for each experiment. Data represent means ±SD and were analyzed using Student’s *t* test; significance levels: *p < 0.05; **p < 0.01.

To investigate the functional role of the RYSV P6 protein during the early stage of viral infection in VCMs, we transfected cells separately with *dsP6* and *dsGFP* and then incubated them with the RYSV inoculum. To rule out the possibility that small RNAs derived from *dsP6* could directly target RYSV genomic RNA and anti-genomic RNA, we amplified a 300-bp fragment from the 3′UTR of the RYSV genome, which is critical for the replication of RYSV genome, and used it to prepare *ds3′UTR*. We also transfected the *ds3′UTR* into cultured *N. cincticeps* cells and then performed RYSV inoculation as a control. At 3 dpi, we immunolabeled the infected VCMs treated with different dsRNA with antibodies specific to N protein, which represents RYSV viroplasm. Confocal microscopy results showed that far fewer fluorescent foci (around 25% of nuclei fluoresced) representing RYSV infection were observed in the cells transfected with *dsP6*. By contrast, abundant fluorescent foci appeared in RYSV-infected cells treated with *dsGFP* (60%) or with *ds3′UTR* (55%) (Figure 6A). These results suggested that the small RNA produced from *ds3′UTR* did not affect the accumulation of RYSV genomic RNA and that the P6 protein plays an important role in the establishment of the viroplasm.

We further assessed the effects of RNAi induced by dsRNAs on the viroplasm in RYSV-infected VCMs (Figure 6C) by qRT-PCR using primers specific to the *N*, *P*, *P6*, and *M* genes. Consistent with the results of the immunofluorescence assay, *ds3′UTR* treatment did not affect the levels of *N*, *P*, and *M* transcripts (Figure 6B). Meanwhile, the *N*, *P*, *P6*, and *M* transcript levels were significantly reduced after *dsP6* treatment, as compared with dsGFP treatment, in RYSV-infected VCMs. Together these results suggest that knockdown of *P6* interfered with the establishment of the viroplasm, which in turn reduced RYSV infection (Figure 6C).

## Discussion

Cultured leafhopper and planthopper cell lines have played an important role in the investigation of insect-transmitted rice viruses, especially reoviruses and rhabdoviruses. However, the lack of an efficient transient expression vector greatly impairs their application. In this study, we established and optimized the first leafhopper cell transient expression vector by cloning and analyzing the native promoter of the leafhopper *cytoplasmic actin* gene. Our *actin*-promoter-based vector was able to drive expression of GFP in various cell lines, including leafhopper and Sf9 cells. We also found that the expression efficiency of the exogenous protein was improved by the combination of the *Hr5* enhancer and knockdown of *Dicer2* transcription. Using our new transient expression vector, we determined for the first time the cellular localization of the proteins of a rice rhabdovirus, RYSV, in leafhopper cells. Through confocal microscopy, electron microscopy, and RNAi, we demonstrated that the RYSV-encoded protein P6 is a component of the RYSV viroplasm and plays an important role in RYSV infection of its insect vectors.

As cell culture technology has matured, insect cell lines derived from more than 100 insect species have been established (Lynn, 2001). However, most lack useful promoters for exogenous protein expression. In particular, there has been no transient expression vector for Hemiptera insect cell lines. Since we created the first brown planthopper (*Nilaparvata lugens*) cell line in 2014 (Chen et al., 2014), great efforts have been made to construct an effective expression vector that works in these Hemiptera insect cell lines. We have tested several commercial insect cell expression systems in Hemiptera insect continuous-culture cells, including the Lepidopteran nucleopolyhedrovirus *immediately early 1* gene promoter (*IE1*)-based vector, a human CMV *immediate early* promoter-based vector, and the Bac-to-Bac baculovirus expression system, which are currently considered the most efficient systems for expressing target proteins in various insect cell lines. Unfortunately, none of these vectors works in leafhopper and planthopper cells, due to the high specialization of cultured cells from these species. When two effective promoters of the leafhopper actin gene, the Nl_act3 and Nc_act1 promoters, were evaluated, it was found that only one, the Nc_act1, worked in s2 cells, but both failed to express exogenous genes in cultured leafhopper cells (Qian et al., 2016). A comparison of the Nc_actin1 promoter (1926 bp) with our *cytoplasmic actin* promoter (2026 bp) showed that their nucleotide sequences are almost identical, except for an extra 100 nucleotides in the 5′ end of our *cytoplasmic actin* promoter, which we think are necessary for the efficient expression of target protein in leafhopper cell culture lines. Our vector has a wide range of applications, including expression of foreign proteins in leafhopper and even Sf9 cells.

Several factors affect the level of heterologous protein expression and the transfection efficiency in insect cells. The introduction of the expression vector into cultured insect cells is the first critical step in obtaining efficient expression of exogenous proteins. Liposome-mediated transfection (lipofection), first reported by Felgner et al. (1987), is one of the most widely used methods and provides improved transfection efficiency in several widely used insect cell culture lines, including *Bombyx mori* (Bm5), *S. frugiperda* (Sf9), and *Lymantria dispar* (IPLB-LdEp, IPLB-LdEIta, IPLB-Ld652Y) cell lines (Trotter and Wood, 1996; Gundersen-Rindal et al., 2000; Whitt et al., 2001), due to liposomes’ low immunogenicity, low toxicity, ease of production, and potential for transferring large pieces of DNA into cells. In our study, we examined three different liposome reagents (Cellfectin II, Lip2000, and Lip3000) that have all been reported to produce consistent and efficient transfection in different cultured insect cell lines. Our results indicated that 2 μg plasmid in 4 μl Cellfectin II provided the greatest transfection efficiency, with only minor cell damage, in our cultured leafhopper cells. The enhancer element in the promoter sequence is believed to improve the transcription of the exogenous gene (Brown et al., 2015; Wang et al., 2018a). We also found that placing the *Hr5* viral enhancer element upstream from the sequence of our *cytoplasmic actin* promoter resulted in optimized expression of GFP in leafhopper cells, indicating that the *Hr5* enhancer acts on the leafhopper *actin* promoter. Importantly, knocking down the *Dicer2* gene further improved the expression efficiency of the GFP in transfected leafhopper cells, implying that the RNAi pathway also affects the expression efficiency of exogenous genes in this system. Dicer-2 in arthropods contribute to the production of siRNA via cleaving endogenous long dsRNA including transposon transcripts, partially self-complementary hairpin RNAs, and mRNA transcription (Kandasamy and Fukunaga, 2016). Several studies showed that the knock-down of Dicer-2 significantly inhibits the efficiency of RNA silencing in insects (Aliyari and Ding, 2009; Lan et al., 2016). However, the specific function of Dicer-2 in the expression of exogenous proteins remains unclear, which needs to be investigated.

Taking advantage of our new *actin* promoter-based transient vector, we first determined the cellular localization of RYSV-encoded proteins in leafhopper cells. RYSV-encoded P6 has been demonstrated to be an RNA silencing suppressor in plant cells, which inhibits the amplification of silencing signals by interfering with the activity of RDR6 (Guo et al., 2013). The previous investigation suggested that P6 appears to be a structural protein of RYSV (Huang et al., 2003). Our confocal microscopy observations indicated that RYSV-encoded P6 accumulated in the nuclei of vector-transfected leafhopper cells. The morphological characteristics of the P6-formed structures we observed during RYSV infection are identical to those of RYSV viroplasm, which implies that P6 also has a novel function associated with RYSV viroplasm. Our *in vitro* assay demonstrated that P6 interacts with N and P. Furthermore, the coexpressed P6 and N or P were colocalized in the nuclei of insect vector cells, indicating that P6 may be a component of RYSV viroplasm, which confirms the result of Huang *et al* and suggests that P6 protein like other two structure proteins, N and P, may involve in the establishment of the viroplasm. However, our results did not rule out the previously proposed function of P6 as an insect RNA silencing suppressor.

The low expression efficiency (around 20% maximum) of our transient expression vector remains an urgent issue to be addressed. More work will be required to optimize the vector for higher expression efficiency. For example, a large double-stranded DNA virus was identified through a small RNA deep-sequencing analysis of leafhopper (Cheng et al., 2014), and we expect that a promoter derived from the genome of this native DNA virus may be suitable for the establishment of a more efficient expression vector. In addition, with the *N. cincticeps* genome sequencing program completed, more efficient native promoters of *N. cincticeps* for exogenous protein expression should be evaluated in cultured cell lines. Moreover, other methods of DNA transfection, such as electroporation, should be tested in leafhopper cell lines.

## Author Contributions

X-FZ and YX conducted the experiments. X-FZ and TW designed the study and wrote the manuscript. JW, HW, TZ, YZ, and HC assisted in conducting the experiments. X-FZ and TW supervised the experiments overall.

### Conflict of Interest Statement

The authors declare that the research was conducted in the absence of any commercial or financial relationships that could be construed as a potential conflict of interest.
