# Supplementary material for: Exploration of an Actin Promoter-Based Transient Expression Vector to Trace the Cellular Localization of Nucleorhabdovirus Proteins in Leafhopper Cultured Cells
Source: Front Microbiol. 2018 Dec 19;9:3034. doi: 10.3389/fmicb.2018.03034 (PMC6306041; doi:10.3389/fmicb.2018.03034)
Supplement: Supplementary file 1 [file Table_1.DOCX]

**Table S1. Concentrations of plasmids and liposomes tested in NC-Cyto-GFP plasmid transfection assay**

| **Plasmid**  **Reagents**^a^ | **1 μg** | **2 μg** | **4 μg** | **6 μg** |
| --- | --- | --- | --- | --- |
| **Cellfectin II** | +^b^ | ++ (5%) | + | + |
| **Lip2000** | - | - | - | - |
| **Lip3000** | - | - | - | - |

^a^ 4 μl liposomes were used to mix with plasmids

^b^ + indicates that around 2.5% transfected cell show GFP signals. – indicates that no fluorescence signals were observed

**Table S2. Optimization of the inoculation time of the NC-Cyto-GFP construct**

| **Time**  **Plasmids**^a^ | **12 h** | **24 h** | **48 h** | **96 h** |
| --- | --- | --- | --- | --- |
| **NC-Cyto-GFP** | - | - | ++ | ++ |

^a^ 4 μl cellfectin II reagent mixed with 2 μg NC-Cyto-GFP plasmid were used in this assay

**Table S3. Primers used in this study**

| **Primer usage** | **Primer name** | **Primer sequence (5'-3')**^a, b^ |
| --- | --- | --- |
| NC-Cyto-GFP | F-Nc-cytoactin-*Eco*R I | GGG GAATTC ACGCCGCCAAGCTTTTTCCATAT |
|  | R-Nc-cytoactin-*Bam*HI | GGG GGATCC GGTGTTTCAATAAATTAACTGCAA |
| NC-Hr5Cyto-GFP | F-Hr5 | ATCTCGAGCTCAAGCTTCGAATTCCGCGTAAAACACAATCAAGT |
|  | R-Hr5 | AAAAGCTTGGCGGCGTGAATTCACGCGTAGAATTCTACCCGTAA |
| NC-Hr5Cyto-N-GFP | F-RYSV-N-GFP | TTAATTTATTGAAACACCGGATCCATGGCTAATGATAATGTTTC |
|  | R-RYSV-N-GFP | CACCATGGTGGCGACCGGTGGATCTTGGATACATTGGCAAGTTC |
| NC-Hr5Cyto-P-GFP | F-RYSV-P-GFP | TTAATTTATTGAAACACCGGATCCATGTCAGGTAGCGGCAGTGA |
|  | R-RYSV-P-GFP | CACCATGGTGGCGACCGGTGGATCCTGAGTTACATATTTCTGGC |
| NC-Hr5Cyto-P3-GFP | F-RYSV-P3-GFP | TTAATTTATTGAAACACCGGATCCATGGGCGAGGGGAAGAACC |
|  | R-RYSV-P3-GFP | CACCATGGTGGCGACCGGTGGATCAGCTTCCAGATAGACATCAG |
| NC-Hr5Cyto-M-GFP | F-RYSV-M-GFP | TTAATTTATTGAAACACCGGATCCATGCACCTGTACTGCTATGC |
|  | R-RYSV-M-GFP | CACCATGGTGGCGACCGGTGGATCTTCTTTCCATGACAGCATGC |
| NC-Hr5Cyto-G-GFP | F-RYSV-G-GFP | TTAATTTATTGAAACACCGGATCCATGGTTTTAATAATAAAAA |
|  | R-RYSV-G-GFP | CACCATGGTGGCGACCGGTGGATCTAAGGCATTGGTAGCTGTGC |
| NC-Hr5Cyto-P6-GFP | F-RYSV-P6-GFP | TTAATTTATTGAAACACCGGATCCATGTCCAGCCAACAAGAAAC |
|  | R-RYSV-P6-GFP | CACCATGGTGGCGACCGGTGGATCGTCCGGCCAGCCGTTGCACT |
| NC-Hr5Cyto-P6-His | F-RYSV-P6-His-*Bam*HI | CGGGATCCCGATGTCCAGCCAACAAGAAAC |
|  | R-RYSV-P6-His-*Not*I | ATTTGCGGCCGCTTTATTAATCTGGCCAGCCGTTGCACT |
| NC-Hr5Cyto-N-Strep | F-RYSV-N-Strep-*Bam*HI | CGGGATCCCGATGGCTAATGATAATGTTTC |
|  | R-RYSV-N-Strep-*Not*I | ATTTGCGGCCGCTTTATTATTTTTCGAACTGCGGGTGGCTCCAAG  CGCT TTGGATACATTGGCAAGTT |
| NC-Hr5Cyto-P-Strep | F-RYSV-P-*Bam*HI | CGGGATCCCGATGTCAGGTAGCGGCAGTGA |
|  | R-RYSV-p--*Not*I | ATTTGCGGCCGCTTTATTATTTTTCGAACTGCGGGTGGCTCCAAG  CGCT CTGAGTTACATATTTCTGGCTC |

^a^ Underlining represents restriction sites in corresponding primers.

^b^ Shading represents the adaptor sequence for the Gibson assembly cloning method.
